# Analysis of factors influencing expressway speeding behavior in China

**DOI:** 10.1371/journal.pone.0238359

**Published:** 2020-09-28

**Authors:** Zijun Liang, Yun Xiao

**Affiliations:** School of Urban Construction and Transportation, Hefei University, Hefei, Anhui, China; Tongii University, CHINA

## Abstract

Based on the characteristics of expressway driving behavior, a punishment avoidance variable is introduced in this study to modify the theory of planned behavior (TPB), and the analysis model of expressway speeding behavior is improved and verified through survey data. The mechanism of the effects of attitude to behavior, subjective norm, perceived behavioral control, and punishment avoidance on expressway speeding behavior is analyzed. The results show that drivers lack a correct understanding of expressway speeding behavior and that punishment avoidance has a significant effect on expressway speeding behavior. Younger drivers (25–34), men, High income earners, and those who received more penalty points are considered prone to speeding. The study provides valuable contributions to the development of the Chinese version of the expressway speeding analysis model.

## Introduction

Road traffic safety is an important issue of concern to society [1]. Every year, global traffic accidents cause 1.35 million deaths, causing losses that exceed 3% of the gross domestic product of most countries [2]. The traffic safety situation in China is relatively severe, with a high death rate per 100,000 motor vehicles, and there is still much room for improvement in the traffic safety environment. Road traffic is a complex system consisting of people, cars, and roads, and people are the most active and subjective factors. In a prior study, Sayed [3] analyzed Canadian traffic accident data and noted that driver misconduct directly caused 65% of accidents and indirectly caused 90% of traffic accidents.

Speeding behavior is a common driver misbehavior and one of the major causes of traffic accidents [4–6]. 9217 people were killed in traffic accidents caused by speeding in 2017, which represents 26% of the total number of traffic accidents in the United States. The increase in average vehicle speed is directly related to the probability of an accident and the severity of consequences of the accident. For every 1% increase in average speed, the risk of fatal collisions increases by 4%, and the risk of accidents that cause injuries increases by 3% [2]. A study addresses the characteristics and trends of road accidents on a selected stretch of NH-1 between RD 98 km and 148 km, and point that speed is the most critical factor of road safety, and higher speed and speed changes increase the number of accidents and the probability of casualties [7]. The driver's control of the vehicle will be greatly reduced with the increase of vehicle speed, which will lead to the possibility of collision [8, 9].

There are many factors that affect driver's speeding. The geographical location of drivers accounted for about 7.7% of the variability in the likelihood of a driver driving over the posted speed [10]. Drivers do not typically feel nervous about speeding on long and straight-line roads, leading to less vigilance and more speeding, which are the main reasons for traffic accidents [11]. The average night speed of commercial vehicles is higher than the average daytime speed, the ratio of overspending during the day and to overspending at night is more than 5%, the daytime acceleration value is greater than the night acceleration value, and the deceleration value is greater at night than in the daytime [12].

Driver's personal attributes are closely related to speeding behavior [13, 14]. Many drivers are subject to a “time saving bias”, tending to drive at a higher speed [15]. Attitude is the key factor to decide whether the driver is speeding or not [16, 17]. Chinese scholars show that all 6 elasticity values of sex, age, education level, corrected vision, professional driver status, and traffic accident occurrence have low elasticity in relation to driving speed decisions. The elasticity values of driving age and personality have higher elasticity in relation to speed decisions, and their effects are significant [18]. Drivers' satisfaction with the speed limit is the most significant variable that positively affects drivers' compliance with speed limit instructions under conditions of low and high hazard perception [19].

The theory of planned behavior (TPB) provides a theoretical framework to study the relationship between personality and behavior. Jovanović et al. [20] investigated 546 drivers from five local communities in the Republika Srpska with the theory of planned behavior, and analyzed the characteristics of speeding behavior on suburban roads, come to conclusion that personal norm, subjective norm, and affective attitudes were shown to be important variables within the modified TPB in understanding speeding behavior. Zhang et al. [21] found that TPB can be used to explain the causes of drivers' unsafe driving behavior at urban-rural fringe. As for urban roads, competitive driving behavior could be predicted by the combination of attitudes, subjective norm, control of perceived behavior, and social environment through the medium of behavior intention [22]. Zhou et al. [23] examine pedestrians’ self-reported violating crossing behavior intentions by applying the theory of planned behavior.

At present, researches on speeding mainly focuses on the relationship between speeding and accident, and the causes of speeding. Additionally, the application of planning behavior theory in driving behavior mainly focuses on urban roads and ordinary highways, but less on expressways. However, the operation of expressways has the characteristics of full closure, high speed, and large flow, making them quite different from other roads. Few scholars consider mechanisms of expressway speeding behavior from the driver's perspective. Thus, to explain how drivers’ psychology plays a role and generates expressway speeding behavior, this study uses the theory of planned behavior to conduct a questionnaire survey of drivers and analyzes their psychological characteristics, thereby proposing ways to improve expressway traffic safety in China.

## Methods

### Ethical note

This study is based purely on observational data. Before implementing the study, our research plan was discussed by several experts. They believed that the questionnaire would not cause any mental injury to the participants, nor would it have any negative social impact or affect the participant. As a consequence, they agreed that the research plan was scientifically sound and feasible, and comply with laws and regulations in China. In addition, at the beginning of the questionnaire, there is an option: "are you willing and agree to complete this survey?”, all the participants voluntarily decided whether to continue. We obtained informed consent from the participants, informing them that the results of the survey would be used only for academic research and that would not have any negative impact on them. Follow-up research can only be carried out with their permission. The investigation was conducted in a voluntary and anonymous manner.

### Improved model

The TPB was first proposed by Ajzen I to explain the process of decision-making about general behavior. The TPB posits that human behavior is determined by behavior intention, which is in turn affected by three factors: attitudes to behavior, subjective norms, and perceived behavioral control [24]. The three factors determine external factors such as beliefs, attitudes, work characteristics, and personality characteristics.

According to the TPB design methodology, the questionnaire involved behavioral attitudes, subjective norms, perceived behavioral control, behavioral intentions, and speeding behaviors. The questionnaire test items used a Likert 7-level scale divided into 7 levels from 1 to 7, indicating the interviewees' opposition and approval to the situation described in the question. The actual driving behavior of the driver is indicated by the frequency with which the driver answers the questions about speeding.

In the process of improving the TPB, studies have found that adding new variables can increase the explanatory power of the theory. Traffic-related punishment refers to the administrative punishment for the person who violates the rules and regulations, according with the traffic management laws, which is an important factor that restricts driver behavior. Many scholars have analyzed the relationship between traffic punishment and driving behavior, and hold that drivers have strong psychology of avoiding punishment [25, 26].

**Fig 1:**
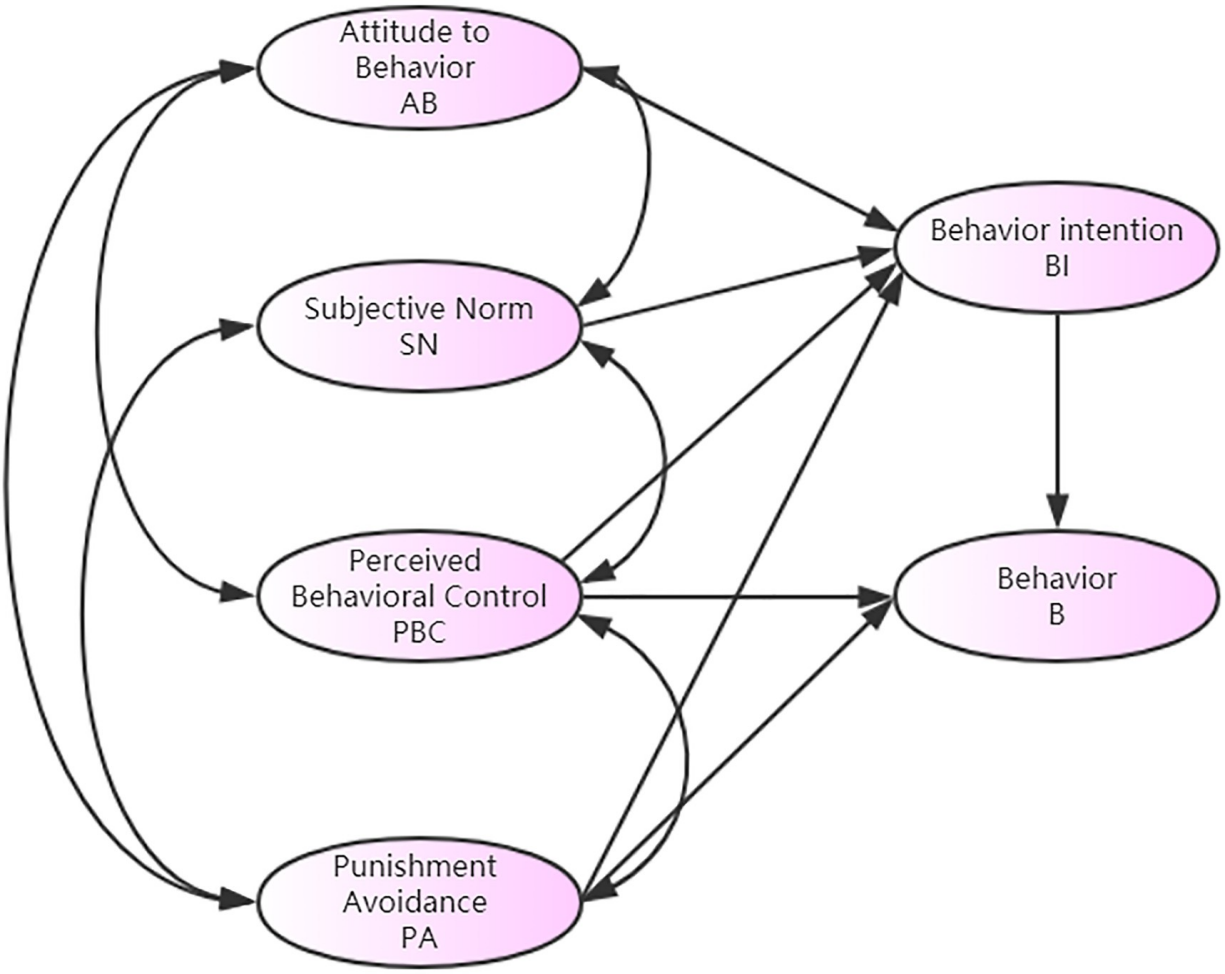
The improved TPB model.

Based on the foregoing theory, this study adds a new psychological factor to the punishment avoidance in the classic model of the TPB. Psychological factors are both independent of and related to each other, together, they affect behavioral intentions, thereby promoting behaviors. At the same time, perceived behavior control and punishment avoidance also influence the behavior itself (Fig 1).

## Questionnaire survey

### Survey method

The study adopts a combination of online surveys and field surveys. A total of 155 online surveys were received, and 109 of them were valid questionnaires. The field survey sites included 111 pairs of expressway service areas in Anhui Province, and the survey objects were all drivers residing in the expressway service area. To improve the accuracy of the survey, the field survey was coordinated by the staff in the service area. Two investigators specifically administered it, 498 questionnaires were collected, 366 of which were valid. The questionnaire was conducted anonymously.

The effective response rate for the online survey was 70.9%, the effective response rate for the field survey was 73.5%, and the response rate was low. The rates were low mainly because in order to improve the effectiveness of the questionnaire, invalid questionnaires were removed for three reasons. First, some questionnaires were completed abnormally, such as by directly checking an entire column of data. Second, the questionnaire indicating that the driver never speeds was considered to have no research value. Third, the questionnaire filled out by the truck driver was eliminated mainly because a truck is affected by the vehicle’s limited technical conditions and it is difficult to reach excessively high speeds; thus, the questionnaire was not useful for this research.

### Questionnaire design

The questionnaire design includes demographic variables and TPB variables. The demographic variables mainly represent basic driver’s information, including age, gender, income, age, and family situation. The item measures for the TPB variables were formulated based on guidance which emphasizes the need to formulate questions for intentions as well as behavior on the basis of target, action, and context.

The extended TPB model mainly consists of five dimensions: attitude to behavior, subjective norm, perceived behavioral control, punishment avoidance, and behavior intention. Each dimension has 3 questions to reduce survey errors. The evaluation uses a 7-point scale ranging from 1 to 7 points. The higher the score, the more the driver agrees with the question. Attitude to behavior mainly tests the driver's opinions on speeding behavior and on whether speeding behavior brings pleasure. Subjective norm specification mainly refers to pressure from society for drivers to speed and mainly involves the views on speeding by family members, people in the car, and friends. Perceived behavioral control mainly indicates a driver's competence to engage in speeding behaviors. The stronger the competence, the more likely that speeding behaviors will occur. Punishment avoidance mainly concerns the drivers’ compliance with traffic policies and regulations. Behavior intention mainly indicates the driver's intention to engage in speeding behavior. The main questionnaire indicators are shown in Table 1.

**Table 1:** Main questionnaire indicators in this study.

**Factor 1 Attitude to Behavior(AB)**
AB1: Do you think that expressway speeding is permissible
AB2: Do you think that expressway speeding is unavoidable
AB3: Do you think that expressway speed limit design is unreasonable
**Factor 2 Subjective Norm(SN)**
SN1: Your family cares whether you are speeding on the expressway
SN2: Passengers in the car care whether you are speeding on the expressway
SN3: Your friends care whether you are speeding
**Factor 3 Perceived Behavior Control(PBC)**
PBC1: When speeding at high speeds, do you feel that it is more difficult to operate the car
PBC2: When overtaking cars at high speeds, how dangerous do you feel it is
PBC3: When overtaking at high speeds, do you feel that you need to concentrate more to control the vehicle
**Factor 4 Punishment Avoidance(PA)**
PA1: Improved technologies such as mobile speed measurement can make me less likely to speed
PA2: Legally binding punishment helps me control my speed
PA3: The warning signs affect my control of speed
**Factor 5 Behavior Intention(BI)**
BI 1: How likely are you to speed when you are in a hurry?
BI 2: Are you accustomed to speeding even if you are not in a hurry?
BI 3: If you know that there is no speed limit in a certain section, are you willing to speed?

### Reliability analysis of the questionnaire

To verify the validity and feasibility of the questionnaire, this study presents an analysis of the survey data. The reliability of the questionnaire was verified by Cronbach’s alpha coefficient (Table 2). The formula for Cronbach’s alpha coefficient is:

$$\alpha =\frac{k}{k-1}\left(1-\frac{\sum s_{i}{}^{2}}{s^{2}}\right)$$ (1)

The above values are all greater than 0.7, indicating that the designed questionnaire has high reliability, and it could be analyzed in the next step.

**Table 2:** Cronbach’s alpha coefficients for the latent variables.

Variable	AB	SN	PBC	PA	BI
**Cronbach’s alpha coefficient**	0.781	0.835	0.961	0.832	0.712

### Validity analysis of the questionnaire

To verify whether the questionnaire can effectively reflect a driver's psychological behavior, KMO analysis and Bartlett's tests were performed by using SPSS 23.0. The results are shown in Table 3. The KMO coefficient is 0.613, which is greater than 0.50, and the Sig value is 0.00, which is less than 0.05. Thus, factor analysis can be performed.

**Table 3:** KMO and Bartlett's test.

Sufficient sampling of KMO metrics		0.613
**Bartlett's spherical test**	**Approximate chi-square**	155
	**df**	57
	**Sig**	0.000

The relationship between various psychological elements of the questionnaire shows that many drivers believe that speeding is unavoidable, family members, passengers and friends oppose drivers’ speeding, and that vehicles are more difficult to control when speeding. Moreover, punitive measures strongly constrain drivers’ speeding. This questionnaire is able to effectively reflect the driving behavior intention and characteristics of driving behavior.

### Model fit analysis

Fitness indexes is used to evaluate whether the model is compatible with the collected data. Fitness indexes is classified into absolute indexes, relative indexes, and adjustment indexes. The problem with an absolute indicator statement is mainly whether the residual or unexplained variation remains after the model adaptation is still perceptible. The connotation of a relative indicator statement is as follows: When explaining a set of observations, what are the advantages of a particular model compared to other possible models?

In the study, the accuracy of the model is tested by the following evaluation indexes: goodness-of-fit index (GFI), adjusted goodness-of-fit index (AGFI), root mean square error of approximation (RMSEA), normed fit index and comparative fit index (CFI). The evaluation criterion for each indicator is shown in Table 4.

**Table 4:** Test of fit of the model (Model fit indexes).

Index	*p*	*GFI*	*AGFI*	RMSEA	NFI	TLI
**Evaluation standard**	P>0.05	>0.90	>0.90	<0.08	>0.90	>0.90
**Value**	0.058	0.910	0.936	0.0769	0.921	0.903
**Degree of fit**	Better	Better	Better	Reasonable	Better	Better

## Results

### Demographics and descriptive variables

**Fig 2:**
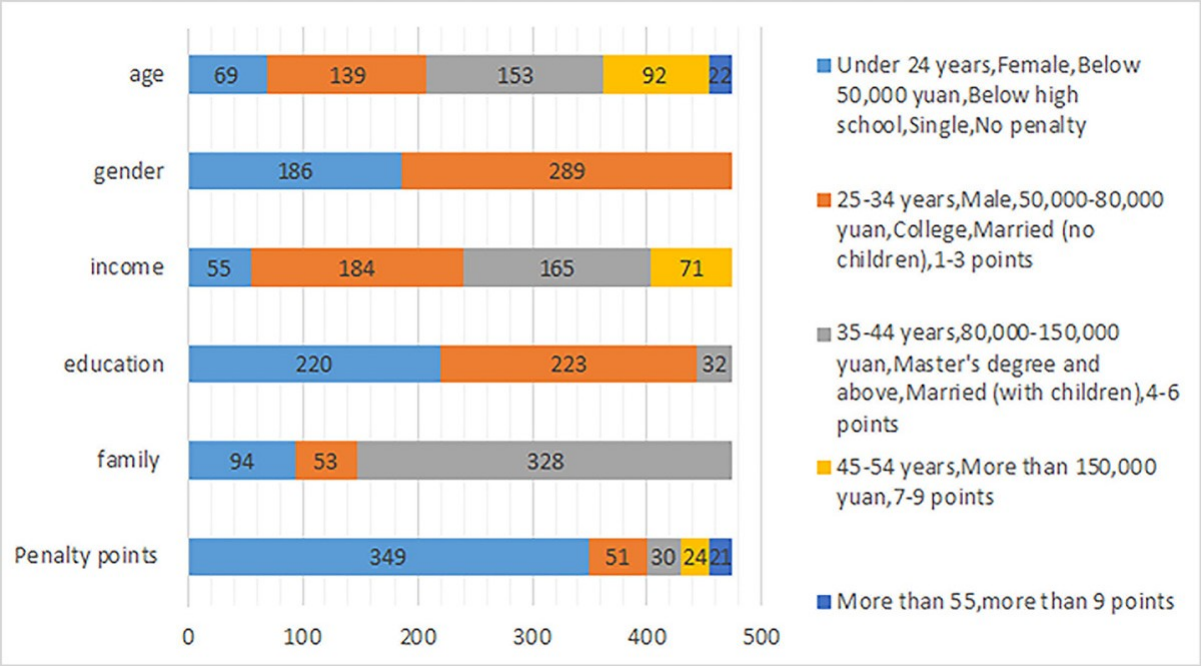
Summary of respondents’ demographic information.

Table 5 and Fig 2 presents the basic information of the drivers who participated in the survey, such as age, income, and driving experience. 289 participants were male and 186 participants were female, and 126 participants (26.5%) received penalty points (M = 0.56; SD = 1.10).

**Table 5:** Demographics and descriptive variables.

Variable	Means (SD)	Range
**Age**	36.45 (10.76)	18–65
**Gender**	0.61 (0.49)	0–1
**Female = 0, Male = 1**		
**Income**	1.53 (0.88)	0–3
**Below 50,000 Yuan = 0**		
**50,000–80,000 Yuan = 1**		
**80,000–150,000 Yuan = 2**		
**More than 150,000 Yuan = 3**		
**Education**	0.60 (0.61)	0–2
**Below high school = 0**		
**College or undergraduate = 1**		
**Master's degree and above = 2**		
**Family**	1.49 (0.80)	0–2
**Single = 0**		
**Married(no children) = 1**		
**Married(with children) = 2**		
**Penalty points in the last year**	0.56 (1.10)	0–4
**No penalty = 0**		
**1–3 points = 1**		
**4–6 points = 2**		
**7–9 points = 3**		
**More than 9 points = 4**		

### Analysis of the influence of personal attributes

The questionnaire included information on drivers' age, income and other personal attributes. To find the correlation between personal attributes and speeding behavior, SPSS 23.0 was used to analyze the responses.

#### Analysis of age differences

Ages was divided into 5 dimensions (Table 6), through analysis of variance, speeding behavior on expressways clearly differs according to age (F = 2.83, p <0.05). The results indicate that speeding behaviors are most likely to occur in groups aged 25–34 and that driving behaviors are more conservative in groups over 55 years of age. In general, individuals who are 25–44 years old have a high average score and thus are considered the "prone to speeding Group."

**Table 6:** Analysis of variance in expressway speeding with different ages.

Variable	Under 24 years	25–34 years	35–44 years	45–54 years	More than 55 years	F	P value
**Speeding behavior**	3.40±0.67	3.78±0.71	3.65±1.43	3.58±0.75	3.23±0.60	2.83	0.02

#### Analysis of gender differences

There is a significant difference between male and female in expressway speeding behavior (Table 7). Male's speeding behavior is reported to be significantly greater than that of women, and men are thus "prone to speeding."

**Table 7:** Analysis of variance in expressway speeding by gender.

Variable	Male	Female	F	P value
**Speeding behavior**	3.71±0.84	3.48±1.11	6.19	0.01

#### Analysis of income differences

Income was divided into 4 dimensions, which has a significant effect on speeding behavior on expressways (Table 8). Speeding behavior is significantly greater for groups with an annual income greater than 150,000 Yuan than it is for other groups. Further analysis shows that higher-income groups have a faster pace of life, higher value of time, and better vehicle grades, which make them prone to speeding. In terms of income, the high-income group is considered to be "prone to speeding."

**Table 8:** Analysis of variance in expressway speeding with different incomes.

Variable	Below 50,000 Yuan	50,000–80,000 Yuan	80,000–150,000 Yuan	More than 150,000 Yuan	F	P value
**Speeding behavior**	3.73±0.71	3.48±0.89	3.65±0.94	3.83±1.27	2.76	0.04

### Analysis of education differences

Educational background was divided into 3 dimensions (Table 9). Analysis of variance shows that there is no significant difference in expressway speeding behavior on the basis of educational background; thus, there is no obvious correlation between educational background and expressway speeding behavior.

**Table 9:** Analysis of variance in expressway speeding with educational background.

Variable	Below high school	College	Master's degree and above	F	P Value
**Speeding behavior**	3.65±0.98	3.59±0.94	3.57±0.88	0.24	0.25

### Analysis of the family situation

Family situation was divided into 3 dimensions (Table 10). There is no significant difference between the family situation and expressway speeding behavior, indicating that this factor has little impact on expressway speeding behavior.

**Table 10:** Analysis of variance in expressway speeding with family situation.

Variable	Single	Married (no children)	Married (with children)	F value	P
**Speeding behavior**	3.68±0.64	3.59±0.83	3.61±1.07	0.23	0.79

### Analysis of differences in penalty points

Penalty points divided into 5 dimensions (Table 11). The results show that penalty points lead to a significant difference in speeding behavior on expressways (F = 4.30, p <0.01). Penalty points involving deductions of 9 points or more are associated with being "prone to speeding."

**Table 11:** Analysis of variance in drivers' expressway speeding with different penalty points.

Variable	No penalty	1–3 points	4–6 points	6–9 points	More than 9 points	F value	P
**Speeding behavior**	3.53±0.93	3.66±1.11	3.79±0.92	3.98±0.98	4.29±0.28	4.30	0.00

### Structural equation model path analysis

**Fig 3:**
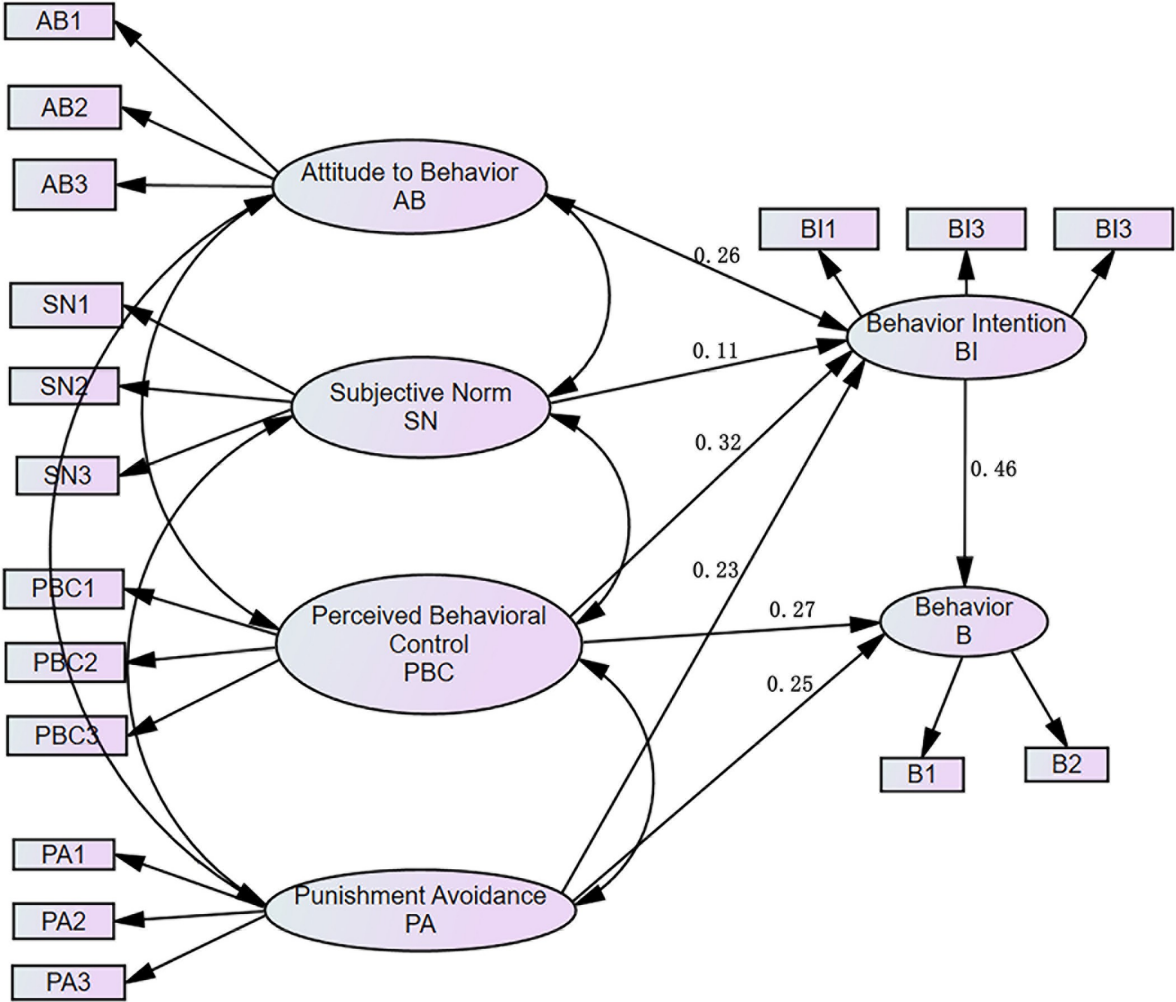
Relationship between the TPB variables.

According to the improved model of TPB, the data of the questionnaire variables were input. To facilitate forward analysis, the scores of subjective norms and penalty variables are positively transformed, and finally, the map of drivers' speeding behavior structural path was obtained (Fig 3).

Behavior intention (BI) = 0.26AB + 0.11SN+ 0.32PBC + 0.23PA

Behavior (B) = 0.27PBC + 0.46BI + 0.25

## Discussion

In this paper, the driving behaviors of expressway were investigated by means of questionnaires with the purpose of exploring the reason of Expressway speeding. Traffic system is a dynamic system composed of human, vehicle, road, environment and other factors. As a random event of this dynamic system, traffic accident is the product of the unbalanced expressway traffic system and the result of the combined effect of multiple factors. Good road driving conditions are easy to induce drivers' speeding behavior. However, due to the fast speed of expressway driving, once traffic accidents happen, they are often serious and malignant, and the accident mortality is also high. Expressway overspeed is still common in China, in the top 10 speeding statistics of Anhui province Expressway, the driver's speed reached 226km / h in February 2019, approaching the speed of high-speed rail [27].

ANOVA of the different personal attributes found that ages, genders, incomes and penalty points were significantly in expressway speeding. Many scholars believe that young drivers are generally adventurous driving style, easy to speeding [28]. However, the results in this study were inconsistent with them, and shows that drivers (M = 3.40; SD = 0.67) younger than 24 are less likely to speed in expressway, which may be due to the lack of driving experience and poor driving skills of young people. Drivers (M = 3.78; SD = 0.70) between 25 and 34 have the highest rate of speeding, and then the proportion of speeding gradually decreases with the increase of age, people (M = 3.23; SD = 0.60) over 55 have the lowest rate of speeding. Male and female have different driving styles, Man's driving risk is far greater than female's [29]. The study shows that Male (M = 3.71; SD = 0.83) are more likely to speed than female (M = 3.48; SD = 1.10) on expressway. Income was significant correlation with speeding behavior, and people (M = 3.82; SD = 1.27) with an annual income of more than 150,000 Yuan have the highest probability of speeding. Penalty points is closely related to driving behavior [30]. The study found that there is a positive correlation between Penalty points and speeding behavior.

According to structural equation model path analysis, the four external potential variables of attitude to behavior, subjective norm, perceived behavior control, and punishment avoidance have significant relationships with behavioral intentions.

In terms of attitude, drivers do not pay enough attention to speeding behaviors. Drivers tend to think that speeding is unavoidable (M = 3.72; SD = 0.87), expressway speeding is permissible (M = 3.43; SD = 1.02), and the speed limit at high speeds is unreasonable (M = 3.98; SD = 0.73). Drivers generally have doubts about the speed limit design of expressways. On the one hand, government must guide drivers to comply with speed limit signs. On the other hand, government should design the maximum speed limit more scientifically, especially on key sections such as bridges and tunnels, to avoid the perceptions of the limit being "prefer low to high" or "sudden higher and then suddenly low" [31].

In terms of subjective norms, driver can perceive social pressure while driving, passengers in the car have the strongest impact on drivers' behavior (M = 4.43; SD = 0.61). Their impact may be the strongest due to their own safety is involved, causing them to promptly remind the driver to stop speeding. The next greatest impact is that of family members (M = 3.73; SD = 0.82), who are more successful in discouraging drivers’ speeding behavior than friends (M = 3.53; SD = 1.10) do. Therefore, it is necessary to emphasize the role of family education, carry out traffic safety education in the community and in family activities, and jointly create a good environment for transportation in order to reduce or even eliminate speeding.

In terms of perceived behavior control, the path coefficient is large (0.32), which indicates that perceived behavior control has a relatively strong impact on behavior, and the driver's ability to control speeding behavior is thus an important factor affecting this behavior.

In terms of punishment avoidance, the path coefficient of behavior avoidance is relatively larger (0.23), which indicates that punishment avoidance has a relatively high impact on behavior intention. Traffic-related punishment therefore has an important effect on restricting drivers' intention to speed on expressways.

In terms of behavior, three potential variables are considered: perceived behavior control, punishment avoidance, and behavior intention. Behavior intention has a direct relationship with the occurrence of behavior, and the effect is significant (0.46), indicating how to reduce speeding intention will be the focus of future work. Perceived behavior control and punishment avoidance can bypass behavior intention and directly affect behavior, and the path coefficient is large. Improving both behavioral control and traffic management policies are effective ways to reduce expressway speeding behavior.

## Conclusion

Speeding is known to be a common driving behavior that affects traffic safety throughout the world. There are many reasons for expressway speeding, among which driver is the most important one. This paper conducted a research on the problem of express speeding in China by using the theory of planned behavior, analyzed the mechanism of the effects of attitude to behavior, subjective norms, perceived behavioral control and punishment avoidance on expressway speeding behavior and to quantify the relationship between the external and internal dependent variables. The addition of the external dependent variable of penalty avoidance helped to improve the TPB model and increase its explanatory power. The paper concluded that individuals who have higher incomes, more Penalty points, male and age between 24–44 were significantly more prone to speeding than other groups, thus, it is necessary to strengthen the management and tracking of this group. Traffic-related punishment has a strong limiting impact on speeding, drivers have strong psychology of avoiding traffic punishment, it is necessary to strengthen police enforcement of traffic.

This study has certain limitations and must be considered when interpreting the results. Because this study is based on driver self-reported data, there is a bias in social expectations. Although participants were guaranteed complete confidentiality and anonymity, and were geographically separated from researchers even during the test, the usual shortcomings of readme questionnaires were inevitable. In future work, improving the measurement technology is of great significance. Therefore, obtaining personal driving records can provide objective results and confirm self-reported information, thereby reducing concerns about potential response bias.

## Supporting information

S1 File The data of this study. (XLSX)
